# Breastfeeding practices among immigrants living in Finland: Results from the FinChildren survey

**DOI:** 10.1016/j.jmh.2024.100283

**Published:** 2024-11-02

**Authors:** I Muhumed, J Meinilä, R Klemetti, FA Adebayo, SM Virtanen, M Erkkola

**Affiliations:** a Department of Food and Nutrition, University of Helsinki, Helsinki, Finland; b Finnish Institute for Health and Welfare, Helsinki, Finland; c Department of Public Health and Welfare, Finnish Institute for Health and Welfare, Helsinki, Finland; d Faculty of Social Sciences, Unit of Health Sciences, Tampere University, Tampere, Finland; e Tampere University Hospital, Research, Development and Innovation Center, Tampere, Finland; f Tampere University Hospital, Wellbeing Services County of Pirkanmaa, Tampere, Finland

**Keywords:** Breastfeeding, Immigrants, Sociodemographic factors, Ethnic groups, Infant feeding

## Abstract

**Background:**

Breastfeeding is a cornerstone of child health and survival as it provides crucial, non-replaceable nourishment necessary for infant's growth and development. Immigration has been shown to influence breastfeeding particularly among immigrants from low- and middle-income countries. Our aim was to examine breastfeeding practices and sociodemographic characteristics of Somali-, Arabic-, and Russian-speaking in comparison with Finnish-speaking mothers.

**Methods:**

We analyzed data from 5348 mothers with infants who participated in FinChildren survey conducted in 2020. Univariate and multivariate analysis were used to estimate the association between maternal origin and breastfeeding practices by comparing immigrant mothers with native-born mothers.

**Results:**

Somali-/Arabic-speaking mothers were younger, less educated and had higher BMI than Russian- and native Finnish-speaking mothers. Proportions of exclusive breastfeeding at 4–5 months of age were lowest among Somali-/Arabic-speaking mothers (21 %) compared to native-born (49 %) and to Russian-speaking mothers (52 %). Again, Somali-/Arabic-speaking mothers had the highest proportions of mixed feeding (66 %) compared to native Finnish-speaking (38 %) and Russian-speaking mothers (32 %). Being a Somali-/Arabic-speaking mother decreased the odds of exclusive breastfeeding five times (OR 0.20, 95 % CI 0.10–0.45) and quadrupled (OR 4.0, 95 % CI 2.18–7.37) the odds of mixed feeding at 4–5 months of age.

**Conclusion:**

Even though the number of immigrant mothers was low, this study suggests that maternal origin is a significant predictor of suboptimal breastfeeding independent of sociodemographic and antenatal characteristics. There is a need for culturally sensitive interventions to promote breastfeeding among these groups.

## 1 Introduction

Immigration is a growing global phenomenon. Currently, there are 281 million international migrants globally, corresponding to 3.6 % of the world's population (International Organization for Migration 2022). In Finland, the number of immigrants has been increasing annually, with 50,000 new immigrants arriving in 2022 alone (Statistics Finland 2022). This represents a significant increase compared to previous years, when Finland typically received between 29,000 and 36,000 immigrants annually (Statistics Finland 2022). Somali- and Arabic-speaking communities are part of the largest foreign-born populations in Finland (Statistics Finland 2023). According to the latest statistics, immigrant women who speak Somali or Arabic tend to have higher birth rates than native-born mothers. Between 2019 and 2022, the total fertility rate for Somali and Iraqi women was 3.1 and 2.5, respectively, while the rate for native Finnish-speaking women was 1.5 (Statistics Finland 2023).

Breastfeeding (BF) is a universally accepted means of providing optimal nutrition, which promotes infant growth, survival, and development (Dennis et al., 2019). Therefore, exclusive breastfeeding is recommended for the first six months by the World Health Organisation (WHO) and four to six months by the Finnish Nutrition Council followed by continued breastfeeding and the gradual introduction of appropriate complementary foods (World Health Organization (WHO) 2021; Finnish Food Authority, 2024).

Breastfeeding is influenced by multiple factors including maternal sociodemographic factors, social and cultural factors among others (Zhou et al., 2020). There is evidence to show that migration from lower income countries into high income countries may have negative impacts on initiation, exclusivity, and duration of breastfeeding (Dancel et al., 2015; Tyler et al., 2014). This has been observed among South Asians migrating to the UK and Hispanics migrating to the USA (Anderson et al., 2005). Research highlights the complex interplays of factors that may influence breastfeeding of immigrant mothers, many of whom face multiple layers of challenges owing to their migration status, ethnicity, sociodemographic and cultural differences (Dennis et al., 2012). In the Nordic countries, a higher maternal education, being in the labour market, and parity seem to positively influence the breastfeeding practices of immigrant mothers (Grewal et al., 2015; Busck-Rasmussen et al., 2014).

In Finland, for the first time, we are examining how maternal origin and sociodemographic characteristics are associated with breastfeeding practices. This study produces a novel information on immigrant breastfeeding and also may contribute to developing targeted strategies to improve breastfeeding outcomes among immigrants.

## 2 Material and methods

### 2.1 Study setting and context

This study is based on an existing data from the FinChildren (in Finnish: FinLapset) survey. The FinChildren survey is a cross-sectional survey carried out nationwide concerning infants aged three to six months and children aged four years and their families. Launched in 2017, the survey aims to monitor the health, welfare and service experiences of young children and their families living in Finland. All mothers and fathers with babies born between 25.11.2019–16.2.2020 and between 20.4.-12.7.2020 were identified by the Digital and Population Data Services Agency (DPDSA) database and invited to the FinChildren survey (Terveyden ja hyvinvoinnin laitos 2024). Data collection was conducted through a postal survey addressed to parents. Parents’ native language was available in the DPDSA database, and the parents had the opportunity to respond to the questionnaire in Finnish, Swedish, Northern Sámi, English, Russian, Somali, or Arabic. The questionnaire forms are available via The Finnish Institute of Health and Welfare (THL) website (thl.fi/finlapset).

A total of 17,964 mothers and 16,112 other parents were invited to participate in the survey. Data were collected as six separate samples starting on 12th March 2020, in which an invitation letter was sent to the parents in the first sample. The parents were approached between 12 March and 8 July 2020 (spring samples) and between 5 August and 8 December 2020 (autumn samples). Each sample included the data of the parents of babies born in a given 4-week period. The parents were first approached when the youngest infant in the sample was 12 weeks and the oldest 16 weeks old. The sampling criteria were defined as the data of the parents of all babies born during the examined periods with a permanent address. The data included the baby's personal identity code and the parents’ names, address, the year and country of birth, sex, marital status, and mother tongue. All responses returned by 12 January 2021 were included in the research data. At this point, the oldest baby in the sample was 6 months. Additional information about the data collection can be found at (thl.fi/finlapset).

A total of 8977 mothers (50 % of those invited) and 5843 other parents (36 % of those invited) participated in the survey. To expand the content, the survey data of mothers (*N* = 8977) were linked to the Finnish Medical Birth Register using the child's personal identity code when data collection was completed.

The present study focuses on Somali-, Russian-, and Arabic-speaking mothers since these immigrant groups are part of the largest foreign-born populations in Finland (Statistic Finland, 2019). In total, 7508 (83.6 %) native-born mothers, 180 (2.0 %) Russian-speaking parents, 81 (0.9 %) Arabic-, and 29 (0.3 %) Somali-speaking parents participated in the survey. Native-born mothers formed the reference group. Other immigrant mothers (*n* = 802), representing 75 nationalities with a very few participants from each nationality, were excluded from the analysis.

### 2.2 Outcome variables

We had two dependent variables in our study: exclusive breastfeeding and mixed feeding. Based on WHO's classification of breastfeeding, exclusive breastfeeding refers to infants who receive only breastmilk with no additional foods and/or drinks including water. However, the infants may receive vitamin and/or mineral supplements (WHO 2023). In our survey, participants were asked the infant's feeding method at the time when mothers completed the survey. In the present study, exclusively breastfed infants were regarded as infants who were receiving only breastmilk when their mothers answered the survey questionnaire. Mixed feeding refers to infants receiving breastmilk at the time of data collection regardless of other complementary foods or drinks received. Mixed breastfeeding variable was categorized as (mixed feeding) or “other” and exclusive breastfeeding variable as “Yes” (exclusively breastfed) or “No” (other).

As the data used was cross-sectional data with responses collected at different time points, the association between outcome variables and independent variables were examined when infants were four to five months of age (3.5 to 5.4 months). The lower cut point is in line with the Finnish Nutrition Council's exclusive breastfeeding recommendations until 4–6 months of age (THL, 2019). These cut points were also chosen to enable feasible comparison of the data from the immigrant mothers and that from the native Finnish-speaking mothers. Due to lower numbers of participants, analysis on breastfeeding practices at six months was not feasible to conduct.

### 2.3 Exposure measure

In this study, maternal origin was determined by using mother's first language. Immigration status was defined as speaking Somali, Russian or Arabic, not Finnish, as the first language. Personal data of mothers were extracted from the Digital and Population Data Services Agency databases.

### 2.4 Predictors

Evidence shows that our selected predictors: older maternal age, higher education, employment before pregnancy, and partner support are positively associated with breastfeeding initiation and duration (Cohen et al., 2018; Santana et al., 2018). Maternal education data (primary/secondary education, university degree, postgraduate degree) and employment status before pregnancy (employed or unemployed) were from the FinChildren survey's self-reported questionnaire. Maternal age data (< 25, 25–29, 30–34, ≥35) and marital status data (married/living together, not in a relationship) were extracted from the Finnish Medical Birth Register. The analysis were adjusted for confounding factors identified in previous studies (Shi et al., 2021; Amir and Donath, 2007; Erkkola et al., 2010): maternal pre-pregnancy BMI (self-reported at the first antenatal visit, categorized as <20, 20–24.9, 25–29.9, ≥30), smoking during pregnancy (yes/no), parity (0, 1, ≥2 children), gestational age (<37, 37–40, >40 weeks), birth weight, and mode of delivery (caesarean/other) sourced from the Finnish Medical Birth Register, and for maternal feeding intention (breastfeeding/mixed feeding/do not know/did not plan) obtained from the FinChildren survey.

### 2.5 Statistical analysis

Sociodemographic, obstetric, infant, and breastfeeding characteristics were described for the selected study populations: Somali-, Russian-, Arabic-, and Finnish-speaking groups. In our analysis, we combined the Somali- and Arabic-speaking groups into a single study subpopulation, due to their limited participant numbers and also due to the close cultural and religious similarities of these groups (European External Action Service 2021). We used chi-square tests to compare categorical variables and one-way ANOVA for comparing group means. For breastfeeding variables, we compared exclusive vs not exclusive breastfeeding and mixed feeding vs other.

Multiple logistic regression analysis was fitted to estimate the association of maternal origin and sociodemographic characteristics with exclusive breastfeeding and mixed feeding. In the crude model, association between each independent variable and dependent variable was examined one at a time (crude model). In the adjusted model all sociodemographic variables and confounders were entered to the model.

The results from the logistic regression analysis are presented with both crude and adjusted odds ratios and 95 % confidence intervals. A *p*-value of less than 0.05 was considered the threshold for statistical significance. All statistical analyses were performed using Statistical Package for Social Sciences (SPSS), version 23.0.

## 3 Results

A total of 6150 mothers with infants aged between 4 and 5 months answered the survey. After excluding data from mothers who spoke a language other than Finnish, Russian, Somali, or Arabic as their first language, (*n* = 802), 5147 (96 %) native Finnish-speaking mothers, 123 (2.3 %) Russian-speaking mothers and 78 (1.5%) Somali-/Arabic-speaking mothers with 4–5 months old infants participated in the survey.

In general, Somali-/Arabic-speaking mothers were more likely to be younger, less educated, and unemployed (Table 1). These mothers also had higher BMI compared to native-born mothers. Almost one-third of these mothers planned mixed feeding their infants during pregnancy.

**Table 1:** Descriptive analysis of antenatal and sociodemographic characteristics.

Characteristics	Finnish-speaking *n* = 5147	Russian-speaking *n* = 123	Somali-/Arabic-speaking *n* = 78	P value
Maternal characteristics				
Age				
<25	568 (11.0)	8 (6.5)	18 (23.1)	
25–29	1474 (28.6)	22 (17.9)	25 (32.1)	< 0.001*
30–34	1824 (35.4)	48 (39.0)	19 (24.4)	
≥ 35	1276 (24.8)	43 (35.0)	16 (20.5)	
BMI before pregnancy ( kg/m^2^)				
< 20	163 (3.2)	12 (9.8)	-	
20–24.9	2437 (47.3)	56 (45.5)	26 (33.3)	
25–29.9	1214 (23.6)	21 (17.1)	24 (30.8)	< 0.001*
> 30	801 (15.6)	12 (9.8)	18 (23.1)	
Education				
Primary/secondary school	2038 (39.6)	46 (37.4)	53 (67.9)	
University degree	1787 (34.7)	20 (16.3)	14 (17.9)	< 0.001*
Postgraduate degree	1313 (25.5)	57 (46.3)	7 (3.8)	
Marital status				
Married/living together	5031 (97.7)	118 (95.9)	65 (83.3)	< 0.001*
Not in a relationship	106 (2.1)	4 (3.3)	8 (10.3)	
Missing	8 (0.2)	1 (0.8)	5 (6.4)	
Work before pregnancy				
Employed	4055 (78.8)	74 (60.2)	6 (7.7)	
Unemployed	1088 (21.1)	49 (39.8)	70 (89.7)	< 0.001*
Smoking during pregnancy				
Yes	340 (6.6)	12 (9.8)	-	0.02*
No	4494 (87.3)	101 (82.1)	69 (88.5)	
Parity				
0	1887 (36.7)	44 (35.8)	19 (24.4)	
1	1570 (30.5)	35 (28.5)	16 (20.5)	0.001*
>2	1690 (32.8)	44 (35.8)	43 (55.1)	
Antenatal feeding intention				
Breastfeeding	3626 (70.4)	91 (74.0)	52 (66.7)	
Mix/formula	979 (19.0)	23 (18.7)	23 (29.5)	0.02*
Don't know/didn't plan	495 (9.6)	7 (5.7)	<5 (2.6)	
Offspring characteristics				
Birth weight (g)				
M (SD)	3552 (526)	3588 (493)	3445(493)	0.16Ω
Gender				
Boys/Girls	2450/2296	61/53	41/37	
Gestational age				
< 37	475 (9.2)	6 (4.9)	5 (6.4)	
37 – 40	3421 (66.5)	78 (63.4)	56 (71.8)	0.22*
> 40	1237 (24.0)	37 (30.1)	17 (21.8)	
Mode of delivery				
Caesarean	881 (17.1)	21 (17.1)	20 (25.6)	0.14*
Other	4261 (82.8)	100 (81.3)	58 (74.4)	

⁎ Chi-square test was used to compare groups

Ω Means of the groups were compared using Anova

### 3.1 Breastfeeding practices at four to five months

Table 2 summarises breastfeeding practices of immigrant and native-born mothers at four to five months of age. Both the duration of exclusive breastfeeding and the total duration of breastfeeding varied significantly depending on the mother's origin. Somali-/Arabic-speaking mothers had the lowest prevalence of exclusive breastfeeding, with only 21 % of their infants being exclusively breastfed, while the number was 49 % for native Finnish-speaking mothers (*p* < 0.001) and 52 % for Russian-speaking mothers (*p* = 0.60). With regard to mixed feeding, Somali-/Arabic-speaking mothers had the highest proportion (66 %), while the proportion was 38 % of native-born mothers (*p* < 0.001). Conversely, Russian-speaking mothers had the lowest proportion of mixed feeding (32 %), a difference which was not statistically significant compared to native-born mothers. On the other hand, highest proportion on no breastfeeding was observed amongst Russian-speaking mothers (16 %) while the numbers were 13 % and 12.9 % for native Finnish-speaking mothers and Somali-/Arabic-speaking mothers respectively. However, this difference was not statistically significant.

**Table 2:** Breastfeeding practices of immigrant and native Finnish mothers at 4–5 months of age.

Study groups	Exclusive Breastfeeding n (%)	Mixed Feeding n (%)	No Breastfeeding n (%)
Finnish-speaking	2186 (49.1)	1686 (37.9)	698 (13.0)
Russian-speaking	49 (52.1) (*p* = 0.60)	30 (31.9) (*p* = 0.24)	15 (16.0) (*P* = 0.44)
Somali-/Arabic-speaking	13 (21.0) (*p* < 0.001)	41 (66.1) (*p* < 0.001)	8 (12.9) (*P* = 0.98)

Differences between groups were determined using Chi-square and Fisher's exact tests.

Missing data: Finnish-speaking: 14 %, Russian-speaking: 24 %, Somali-/Arabic-speaking: 21 %.

### 3.2 Factors associated with exclusive breastfeeding and mixed feeding at 4–5 months of age

In the regression models fitted, we found that maternal origin was a significant predictor of suboptimal breastfeeding practices. Being a Somali-/Arabic-speaking mother decreased the likelihood of exclusive breastfeeding both in the crude (OR 0.27, 95 % CI 0.15–0.50) and in the adjusted models (OR 0.20, 95 % CI 0.10–0.45) (Table 3). Additionally, it was observed that infants born to Somali-/Arabic-speaking mothers were more likely to be mixed fed compared to those born to native Finnish-speaking mothers (Table 3). The likelihood of practicing mixed feeding was tripled (OR 3.2, 95 % CI 1.88–5.43) and quadrupled (OR 4.0, 95 % CI 2.18–7.37) in the crude and adjusted models, respectively, when the mother was Somali-/Arabic-speaking (Table 3). Having a Russian-speaking mother was not associated with the breastfeeding practices examined.

**Table 3:** Factors associated with exclusive breastfeeding^¶^ and mixed feeding π at 4–5 months of age.

	Exclusive breastfeeding		Mixed feeding	
	OR (95 % CI)	AOR (95 % CI) ᵻ	OR (95 % CI)	AOR (95 % CI) ᵻ
**Maternal origin**				
Native Finnish	Ref	Ref	Ref	Ref
Russian-speaking	1.12 (0.75, 1.69)	1.04 (0.63, 1.73)	0.76 (0.49, 1.19)	0.81 (0.49, 1.33)
Somali-/Arabic- speaking	0.27 (0.15, 0.50)	0.20 (0.10, 0.45)	3.20 (1.88, 5.43)	4.01 (2.18, 7.37)

*Sociodemographics*				

**Educational level**				
Primary/secondary	Ref	Ref	Ref	Ref
University degree	1.89 (1.66, 2.15)	1.63 (1.38, 1.93)	0.93 (0.82, 1.06)	0.91 (0.77, 1.07)
Postgraduate	1.86 (1.63, 2.14)	1.53 (1.26, 1.85)	1.02 (0.89, 1.17)	0.97 (0.1, 1.175)
**Maternal age**				
<25	Ref	Ref	Ref	Ref
25–29	1.83 (1.49, 2.25)	1.62 (1.25, 2.10)	1.08 (0.88, 1.32)	1.03 (0.81, 1.32)
30–34	1.99 (1.43, 2.43)	1.59 (1.21, 2.08)	1.08 (0.88, 1.31)	1.07 (0.83, 1.38)
≥ 35	1.81 (1.47, 2.23)	1.44 (1.08, 1.93)	1.18 (0.96, 1.45)	1.20 (0.90, 1.58)
***Employment status***				
Unemployed	Ref	Ref	Ref	Ref
Employed	0.95 (0.84, 1.08)	0.81 (0.68, 0.97)	1.18 (1.03, 1.35)	1.22 (1.02, 1.45)
***Marital status***				
Not in a relationship	Ref	Ref	Ref	Ref
In relationship/Living together	1.72 (1.16, 2.55)	1.15 (0.70, 1.90)	1.27 (0.85, 1.89)	1.52 (0.93, 2.47)

In this model, exclusive breastfeeding at 4–5 months of age coded as follows: 0: not exclusively breastfed 1: exclusively breastfed.

π In this model, mixed feeding at 4–5 months of age coded as follows: 0: Other 1: mixed feeding.

ᵻ Adjusted for BMI, antenatal feeding intention, smoking, mode of delivery, gestational age and parity.

Higher maternal education was positively associated with exclusive breastfeeding in both crude and adjusted models. Being in a relationship was also a significant predictor of exclusive breastfeeding in the crude model, however, in the adjusted model its significance diminished. Younger maternal age and being employed were significant predictors of mixed feeding in both crude and adjusted models.

## 4 Discussion

This is the first study to examine whether and how maternal country of origin is associated with breastfeeding practices in the Finnish context. We examined how maternal origin and sociodemographic characteristics are associated with exclusive breastfeeding and mixed feeding at 4–5 months of age. Suboptimal breastfeeding was more common among Somali-/Arabic-speaking mothers compared to native-born mothers. These mothers were younger in age, less educated, had higher BMI and were more likely to plan mixed feeding during pregnancy compared to native Finnish-speaking mothers. Russian-speaking mothers exhibited sociodemographic characteristics more similar to those of native-born mothers.

Previous research in Europe and North America generally suggests that breastfeeding rates are higher amongst migrant mothers compared to native-born mothers (Dennis et al., 2019; Lisi et al., 2020). However, our study found that Somali-/Arabic-speaking mothers had lower exclusive breastfeeding proportions compared to native-born mothers. Studies conducted in Nordic countries including Denmark and Norway have also found similar results. In Denmark, Danish mothers were more likely to exclusively breastfeed at four months compared to immigrant mothers (Busck-Rasmussen et al., 2014). In Norway, only 7 % and 10 % of infants born to mothers of a Somali and Iraqi origin, respectively, were exclusively breastfed at four months while the number was 48 % for infants born to Norwegian parents according to the Norwegian national survey (Grewal et al., 2015). This could be partly explained by the lower prevalence of exclusive breastfeeding in Somalia and in many Arab countries. For example, exclusive breastfeeding rate at six months is around 25 % in Iraq, 28 % in Syria, 28 % in Saudi-Arabia and 24 % in Qatar (World Bank 2023) (UNICEF Iraq 2021) (Hendaus et al., 2018) (Alissa and Alshareef, 2024). According to the UNICEF's report only one-third of Somali infants are exclusively breastfed at six months of age (UNICEF Somalia 2021).

There is evidence to show that certain maternal sociodemographic characteristics are linked to breastfeeding practices (Dennis et al., 2012; Busck-Rasmussen et al., 2014; Magnano San Lio et al., 2021). For example, younger age and lower educational level have been consistently associated with shorter breastfeeding (Magnano San Lio et al., 2021; Mangrio et al., 2017). This could be attributed to the positive impacts of education on breastfeeding knowledge and adherence to health recommendations (Magnano San Lio et al., 2021) as well as the fact that older mothers are more likely to have breastfeeding experience or that they may have higher education (Dennis et al., 2012). Studies conducted in Denmark and the Netherlands have also shown that Immigrant mothers are more often younger and less educated than native mothers (Busck-Rasmussen et al., 2014; Mangrio et al., 2017) This aligns with our study finding in which Somali-/Arabic-speaking mothers who had inadequate breastfeeding practices were younger in age and had lower educational level compared to native Finnish-speaking mothers.

Aside from sociodemographic factors, breastfeeding could be influenced by the antenatal feeding intention, BMI and mode of delivery (Mangrio et al., 2017; Ibrahim et al., 2023). Mothers who intend to breastfeed during pregnancy, breastfeed more frequently and possess more knowledge about nutrition and diet (Raissian and Su, 2018). Higher BMI increases the risk of C-section deliveries which in turn delays mother/infant skin-to-skin contact, which has been associated with a lower BF initiation (Chen et al., 2018; Watt et al., 2012). A review and meta-analysis on the association between immigration and C-section found higher C-section rates among Somali and Asian women compared to native-born mothers (Merry et al., 2013). The authors concluded that a poor maternal health, a language barrier, higher BMI, and a lack of prenatal care might be the reasons behind the observed variations in C-section rates. These factors taken together could be reflected in our finding whereby Somali-/Arabic-speaking mothers, who had suboptimal BF, were also more likely to plan mixed feeding during pregnancy, had a higher BMI and more often underwent C-section deliveries compared to native Finnish-speaking mothers.

Being a Somali-/Arabic-speaking mother decreased the odds of exclusive breastfeeding and quadrupled the odds of mixed feeding compared to native Finnish-speaking mothers, independent of well-known sociodemographic determinants of breastfeeding. This indicates the existence of other factors that may contribute to the suboptimal breastfeeding practices of these mothers such accessibility of the prenatal care.

In Finland, prenatal and postnatal care services are universally available and free of charge. These services are primarily delivered in Finnish, with availability in Swedish and English. While interpreter services are recommended when there is no common language between parents and healthcare professionals, they are not always available.

The Finnish maternal healthcare system, established in 1940s when Finland had minimal migration, was originally designed for a homogeneous population. Despite ongoing development to accommodate migrants needs, the services remain predominantly oriented toward the mainstream population, which may create barriers for ethnic minority groups. Research indicates that immigrant mothers are more likely to have delays or incomplete adherence to prenatal care (Wahlberg et al., 2013; Downe et al., 2009).

### 4.1 Strength and limitations of the study

To our knowledge, this is the first study examining the association between immigration and breastfeeding practices in Finland. Mothers’ response rates in the FinChildren survey were reasonable (50 %) and based on the reference material acquired from Statistics Finland, the coverage of the research samples was good. Unfortunately, number of immigrant mothers remained low with a lower response rate (Russian-speaking; 43 %, Arabic-speaking; 32 %) compared to native Finnish-speaking mothers (53 %). However, the unique nature of our data lies in combining both survey and register data and including data from foreign-born mothers. Given the known knowledge gap regarding breastfeeding among immigrant mothers, our data provides remarkable insights.

The recruitment method was not the most optimal for reaching the immigrant women. The dataset used in the study consisted of secondary data from the FinChildren survey, that aimed to recruit a nationally representative sample of all new-borns. There is evidence to show that stratified sampling methods are more justified when aiming to recruit participants from ethnic minorities (Al-bannay et al. 2014). Even though the participants were able to respond to the questionnaire in Finnish, Swedish, Northern Sámi, English, Russian, Somali, and Arabic, many other languages were missing. This may have prevented other immigrants who did not speak these languages from participating in the study. We hope that our study paves the way for a more detailed consideration of immigrant groups in the future national surveys.

In the FinChildren survey, participants were approached via postal mails, requiring reading proficiency to complete the questionnaire. This approach potentially excluded most illiterate immigrant mothers. Consequently, our findings may underestimate the actual challenges, as illiterate parents, who might face additional breastfeeding barriers, were likely not represented in the study population.

Another limitation is the cross-sectional nature of data and the reliance on maternal self-reporting for the infant feeding practices and maternal characteristics, including height and weight, which may introduce recall and reporting biases. Data has been collected from a single point of time from each participant. We, therefore, could not conduct any longitudinal analysis which might have been more appropriate for breastfeeding data. However, it is worth noting that this dataset was the only available dataset in the Finnish context that included immigrant mothers and is unique as it includes both survey and birth register data. This for example, enabled us to adjust for the influence of many confounding factors including women's intention to breastfeed, BMI and mode of delivery which were all extracted from the Finnish Medical Birth Register.

## 5 Conclusions

Health inequalities between the children of immigrant and native Finnish mothers begin to emerge already during pregnancy due to maternal sociodemographic disparities, which often influence breastfeeding practices. Even though the number of immigrant mothers was low in this study, the study demonstrates that Russian-, Somali-, and Arabic-speaking mothers were more likely to have suboptimal breastfeeding practices compared to native-born mothers. Furthermore, being a Somali-/Arabic-speaking mother predicted suboptimal breastfeeding practices after adjusting for sociodemographic and antenatal factors. This may be attributed to language barriers or insufficient breastfeeding support received during the pre- and postnatal periods. To ensure successful breastfeeding promotion among these population groups and to reduce health inequalities between the children of immigrant and native-born mothers, sufficient support from healthcare professionals is crucial as recommended by the Baby Friendly Hospital Initiative. Therefore, further research is warranted to investigate whether immigrant mothers receive similar breastfeeding support compared to native Finnish mothers, especially given the growing immigrant population in Finland.

## Ethical approval

Permission to use data was received from the Finnish institute of Health and Welfare (THL). FinChildren survey received ethical clearance from the THL ethical board 10.06.2019 (THL/1535/6.02.01/2019).

Data Availability: FinChildren data are available upon application and payment by the Finnish Social and Health Data Permit Authority, Findata. Findata grants permits for the secondary use of social and health care data and improves data protection for individuals. After granting the permit Findata compiles, combines and pre-processes data and offers tools for analysing. https://findata.fi/en/

## Funding

The first author is a recipient of grant funding from the Finnish Cultural Foundation.

## CRediT authorship contribution statement

**I Muhumed:** Writing – original draft, Methodology, Formal analysis, Conceptualization. **J Meinilä:** Writing – review & editing, Supervision, Methodology, Conceptualization. **R Klemetti:** Writing – review & editing, Methodology, Conceptualization. **FA Adebayo:** Writing – review & editing, Methodology. **SM Virtanen:** Writing – review & editing, Supervision, Methodology, Conceptualization. **M Erkkola:** Writing – review & editing, Supervision, Methodology, Conceptualization.

## Declaration of competing interest

The authors declare that they have no known competing financial interests or personal relationships that could have appeared to influence the work reported in this paper.
